# Gingival Creep Failure: A Viscoelastic Theory of Recession in Thin Periodontal Phenotypes

**DOI:** 10.3390/biology15090685

**Published:** 2026-04-27

**Authors:** Anna Ewa Kuc, Natalia Kuc, Jacek Kotuła, Joanna Lis, Beata Kawala, Michał Sarul

**Affiliations:** 1 Department of Dentofacial Orthopedics and Orthodontics, Wroclaw Medical University, 50-425 Wroclaw, Poland; j_kotula@poczta.onet.pl (J.K.); joanna.lis@umw.edu.pl (J.L.); beata.kawala@umw.edu.pl (B.K.); 2 Faculty of Medicine, Medical University in Bialystok, ul. Kilińskiego 1, 15-089 Bialystok, Poland; nataliakuc.med@gmail.com; 3 Department of Integrated Dentistry, Wroclaw Medical University, 50-425 Wroclaw, Poland; michal.sarul@umw.edu.pl

**Keywords:** gingival recession, gingiva, orthodontics, periodontium, connective tissue, extracellular matrix, biomechanical phenomenon, viscoelasticity

## Abstract

Gingival recession is commonly linked to alveolar bone dehiscence, inflammatory burden, traumatic brushing, or excessive orthodontic forces. However, recession is also observed in some patients despite apparently mild or “biologically acceptable” loading, particularly in thin periodontal phenotypes. Here, we propose the Gingival Creep Failure Theory, a hypothesis-driven conceptual framework in which gingival soft tissues undergo time-dependent viscoelastic deformation (creep) under sustained or repetitive tensile microstrain. Over time, accumulated deformation and microstructural fatigue may reduce recoil capacity and shift the gingival margin apically once tissue-level tolerance is exceeded. Gingival connective tissue is modeled as a fiber-reinforced, fluid-rich viscoelastic composite whose response depends on collagen architecture, cross-linking, proteoglycan-mediated hydration, and vascular support. In thin phenotypes characterized by reduced connective tissue volume and altered extracellular matrix (ECM) organization, creep progression is hypothesized to accelerate, lowering the threshold at which fatigue-related microdamage translates into clinically detectable marginal migration. Evidence from collagenous connective tissue biomechanics supports the plausibility that sub-failure sustained or cyclic loading can produce cumulative deformation and incomplete recovery; however, direct creep–fatigue data for human gingiva remain limited, underscoring the need for targeted validation studies. This hypothesis integrates soft tissue mechanics with periodontal phenotype biology and orthodontic loading patterns and proposes creep and microstructural fatigue as plausible time-dependent contributors to gingival recession in susceptible phenotypes. Because direct in vivo gingival strain and creep–fatigue measurements remain limited, the model should be interpreted as hypothesis-generating and in need of targeted clinical and experimental validation.

## Simple Summary

Gingival recession is usually explained by bone loss, inflammation, traumatic brushing, or excessive orthodontic forces. However, in clinical practice, recession may also appear in patients with thin periodontal tissues even when orthodontic forces seem mild and biologically acceptable. This article proposes a new hypothesis: the gingival margin may slowly move because soft tissues undergo time-dependent deformation under repeated or sustained tensile loading. This process is called creep. In this model, gingival connective tissue behaves like a viscoelastic collagen-rich material. Over time, small repeated strain may accumulate, reduce tissue recoil, and produce microstructural fatigue. Thin periodontal phenotypes may be more vulnerable because they have less connective tissue volume, altered extracellular matrix organization, reduced hydration, and lower mechanical reserve. The proposed theory does not replace established causes of recession but adds a possible time-dependent soft tissue mechanism that may interact with bone morphology, inflammation, brushing trauma, and orthodontic loading. Future studies are needed to directly measure gingival strain, creep behavior, and tissue failure thresholds in human gingiva.

## 1. Introduction

Gingival recession remains one of the most frequently observed complications during orthodontic treatment, yet its fundamental biomechanical cause is still inadequately explained. Traditional models attribute recession to alveolar bone dehiscence, inflammatory burden, traumatic brushing, or excessive orthodontic forces. However, clinical evidence shows that recession often occurs even under mild or biologically acceptable loading, particularly in patients with a thin periodontal phenotype [1,2,3]. This discrepancy suggests that current paradigms do not fully capture the mechanical vulnerability of gingival soft tissues.

In this manuscript, the term “creep” refers to the biomechanical phenomenon of time-dependent deformation under sustained or repetitive loading and not to “creeping attachment” described after mucogingival surgery. Gingival connective tissue is not a purely elastic material but a highly viscoelastic, fluid-rich, collagen-reinforced structure that undergoes time-dependent deformation under load. Viscoelastic tissues exhibit *creep*, a progressive increase in strain under constant or repetitive stress, which can lead to irreversible elongation when the material’s microstructural resistance is exceeded [4,5]. Experimental studies in soft connective tissues demonstrate that small, sustained tensile forces are capable of producing permanent fiber elongation, collagen uncrimping, proteoglycan disruption, and microstructural fatigue even when stress levels remain below the elastic failure threshold [6].

In the thin periodontal phenotype, reduced collagen density, lower cross-linking, diminished hydration, and limited vascular support further accelerate creep progression and decrease the threshold at which structural failure occurs [1,7,8]. These microstructural attributes help explain why recession occurs more frequently and more severely in thin phenotype patients, independent of force magnitude.

What is currently missing is not another list of recession risk factors but a testable mechanical framework capable of explaining why gingival recession may develop under apparently mild or clinically acceptable loading in select periodontal phenotypes. The present hypothesis is intended to address this gap by proposing a time-dependent soft tissue failure pathway and outlining concrete routes for validation, including ex vivo creep testing of gingival specimens, patient-specific biomechanical modeling, serial imaging of marginal displacement, and in vivo estimation of tissue deformation using elastography- or optical coherence tomography (OCT)-based methods.

This article proposes the Gingival Creep Failure Theory as a testable hypothesis, suggesting that recession can reflect time-dependent viscoelastic deformation and fatigue-related loss of marginal stability in gingival tissues under sustained tensile microstrain. We outline key mechanistic assumptions, specify scope and limitations, and provide falsifiable predictions and suggested validation approaches.

### Definitions and Scope

Creep: Time-dependent increase in deformation under sustained or repetitive loading in viscoelastic tissues.

Fatigue (microstructural): Cumulative loss of microstructural integrity (e.g., fibrillar damage, cross-link disruption) with repeated or prolonged loading, potentially leading to incomplete recovery.

Microstrain: Small tissue-level tensile deformation acting along the gingival margin generated by orthodontic/perioral loading (order of magnitude in gingiva remains to be established in vivo).

Creep failure threshold (conceptual): A tissue-level tolerance boundary beyond which deformation becomes progressively less recoverable and clinically detectable marginal migration becomes more likely.

Scope: This framework does not deny contributions of inflammation, brushing trauma, or bone morphology; instead, it proposes a time-dependent soft tissue mechanical pathway that may interact with these factors, especially in thin phenotypes.

For transparency, the present framework distinguishes three evidence levels. First, direct evidence includes measured viscoelastic behavior in collagen-rich connective tissues and periodontal/gingival mechanotransduction findings under load. Second, inferred elements include extrapolation of creep-related deformation logic to gingival margin behavior under orthodontic/perioral loading. Third, hypothetical elements include the proposed in vivo magnitude of sustained tensile microstrain in gingiva, the exact location of a creep failure threshold, and the quantitative interaction between phenotype, inflammation, and osseous support. This distinction is maintained throughout the manuscript.

## 2. Background

### 2.1. Gingival Soft Tissue as a Viscoelastic Material

Gingival connective tissue is a structurally complex, fiber-reinforced, fluid-rich material whose mechanical properties cannot be described by elastic models alone. Instead, it behaves as a nonlinear viscoelastic tissue, exhibiting creep, stress relaxation, hysteresis, and time-dependent microstructural deformation under sustained or cyclic loading [4,5,6,9,10].

These behaviors arise from the composite organization of the tissue: collagen type I and III fibers provide tensile resistance; elastin fibers contribute to recoil; proteoglycans and glycosaminoglycans regulate hydration and lubrication; and interstitial fluid flow governs load sharing and energy dissipation [11,12,13].

Under constant tensile microstrain, viscoelastic soft tissues show progressive elongation over time—a hallmark of creep behavior. This occurs even when applied stress remains below the instantaneous elastic failure threshold. Creep reflects the gradual uncrimping of collagen fibers, sliding of fibrillar planes, disruption of proteoglycan cross-links, and rearrangement of water content within the matrix [5,14]. As these microstructural changes accumulate, the tissue transitions from reversible deformation to permanent set, effectively altering the spatial position of the gingival margin.

The gingival ECM shows similar behavior to tendon, ligament, and dermal connective tissues, where creep-induced microdamage is well-documented [6,15]. Importantly, creep is accelerated in tissues with reduced collagen density, lower cross-linking, or impaired hydration—features characteristic of thin periodontal phenotypes [7,8]. Such tissues dissipate less mechanical energy and undergo strain accumulation more rapidly, making them vulnerable to viscoelastic fatigue even under mild orthodontic loading.

These tissues should be regarded as comparator collagenous systems rather than direct equivalents of gingiva. Gingival tissue is a specialized oral mucosal connective tissue characterized by constant exposure to perioral and masticatory forces, intimate attachment to the tooth–periodontium complex, high vascularity, and a distinct epithelial–connective interface. For this reason, evidence from tendon, ligament, and dermis tissues supports mechanical plausibility but does not substitute for direct gingival validation.

These biomechanical insights form the foundation for the Gingival Creep Failure Theory, which proposes that recession reflects time-dependent viscoelastic failure of gingival tissues rather than simply excessive mechanical force or bone loss alone.

### 2.2. Mechanobiology of Creep

Creep is a defining feature of viscoelastic soft tissues, representing a time-dependent increase in deformation under sustained or repetitive load. Unlike elastic deformation, which is instantaneous and reversible, creep reflects progressive microstructural changes within the extracellular matrix (ECM). In gingival tissues, these changes result from the combined effects of collagen fiber uncrimping, interfibrillar sliding, proteoglycan disassociation, and fluid redistribution within the ECM [4,5,14].

Even low-magnitude tensile forces can induce microstrain sufficient to trigger collagen fiber realignment and elongation when applied repeatedly over time. Strain-enhanced stress relaxation in collagen networks and matrix composition effects can facilitate time-dependent deformation, while glycation-related changes may alter fiber sliding behavior [16,17,18]. Soft tissue biomechanics studies show that sub-failure cyclic loading can cause cumulative deformation, with measurable residual elongation after repeated cycles [8,19,20]. This accumulated microstrain is a plausible mechanical precursor of creep-driven tissue instability.

Collagen fibers in the gingival ECM exist in a natural “crimped” configuration. Under tension, this crimp gradually disappears, reducing the tissue’s ability to recoil. When loading persists, microdamage occurs within fibrils and cross-links, lowering stiffness and accelerating the rate of creep [19,20,21]. Over time, these microstructural changes convert initially reversible deformation into permanent set, altering the position of the gingival margin.

Gingival ECM is highly hydrated, and fluid flow contributes significantly to load transmission and energy dissipation. During sustained tensile load, fluid redistribution decreases the internal resistance of the tissue, promoting additional creep and delayed recovery [14,17,21,22]. Proteoglycan degradation further reduces hydration and lubrication, increasing shear strains between collagen fibers.

For conceptual clarity, we use the classical primary/secondary creep terminology as an analogical descriptive framework. In gingiva, these phases should currently be understood as hypothetical organizational concepts rather than validated tissue-specific creep stages. The creep response has a biphasic nature:

Primary creep—slow, nonlinear deformation as collagen uncrimps.

Secondary creep—accelerated deformation once microdamage accumulates.

Beyond a threshold, gingival tissue loses the capacity to recover and undergoes creep failure, resulting in irreversible marginal migration even in the absence of excessive force. Experiments in ligament and dermal tissue demonstrate that viscoelastic fatigue develops faster in tissues with low collagen density or compromised matrix integrity, matching the characteristics of thin periodontal phenotypes [8,19,23].

Orthodontic appliances frequently impose mild but chronically sustained tensile loads on gingival tissues due to tooth inclination, soft tissue tension, lip pressure changes, and archform modifications [24,25]. Over weeks and months, even “light forces” can produce sufficient microstrain for creep accumulation, explaining why recession may develop despite clinically acceptable mechanics.

The mechanobiology of creep therefore provides an essential framework for understanding gingival instability in thin phenotypes and forms the mechanical foundation of the Gingival Creep Failure Theory.

A major current limitation is that the magnitude and duration of sustained tensile microstrain in human gingiva have not yet been quantified in vivo. At present, the proposed key stimulus remains conceptual rather than measured. Future work should therefore prioritize direct estimation of gingival deformation using patient-specific finite element models constrained by CBCT/intraoral scans, ex vivo tensile testing of gingival specimens, and in vivo approaches, such as ultrasound elastography, OCT-based displacement tracking, or serial digital surface analysis under defined loading conditions. Until such measurements are available, the present model should not be interpreted as providing quantitative clinical thresholds.

To connect time-dependent creep to clinical recession, the relevant biological question is how sustained sub-failure mechanical stimuli are transduced into cellular programs that remodel and weaken the gingival extracellular matrix (ECM). Gingival fibroblasts and resident immune cells sense prolonged mechanical loading through integrin-based focal adhesions, cytoskeletal tension, and mechanosensitive ion channels. These inputs converge on canonical signaling nodes (e.g., FAK/Src-associated pathways, RhoA/ROCK cytoskeletal remodeling, MAPK/ERK and p38 stress signaling, and mechanosensitive transcriptional regulators such as YAP/TAZ) [26,27,28,29].

In parallel, sustained deformation can activate latent TGF-β in the matrix and alter the fibroblast phenotype toward either adaptive repair or maladaptive remodeling depending on load duration and matrix integrity [30]. Importantly, creep is not only a passive structural phenomenon; it changes the cell matrix mechanical microenvironment over time. As collagen uncrimping and fibrillar sliding accumulate, local stiffness, anisotropy, and hydration-dependent friction shift, thereby continuously altering mechanotransduction inputs. This provides a mechanistic basis for why “light” forces sustained for long durations may have outsized biological effects compared with brief higher loads. Sustained mechanical stress can also couple to inflammatory and degradative pathways that directly lower creep resistance. Prolonged fibroblast strain and matrix microdamage can promote sterile inflammatory signaling (e.g., via mechanosensitive NF-κB activation and damage-associated molecular cues), increasing the expression of cytokines and chemokines that recruit or activate inflammatory cells. This inflammatory amplification is clinically plausible in thin phenotypes, where reduced perfusion and limited repair capacity may prolong tissue exposure to inflammatory mediators [31,32,33]. Periodontal-specific support for this mechanism is also provided by studies showing that static tensile strain in human periodontal fibroblasts induces inflammatory mediators and matrix-degrading enzymes, including IL-6 and MMP-8 [34]. Animal studies are consistent with this model: orthodontic loading increased gingival MMP-1 activity and altered Col-I/TIMP expression in dog gingiva, whereas pressure-side rat gingiva showed increased IL-1β, MMP-9, and TIMP-1 expression under orthodontic loading [35,36]. Human clinical studies support the same mechanobiological direction: orthodontic tooth movement is accompanied by transient increases in gingival crevicular fluid IL-1β, IL-8, TNF-α, MMP-9, TIMP-1/2, and MMP-1/MMP-8, together with force-dependent changes in gingival blood flow, supporting the clinical relevance of load-induced inflammatory and matrix remodeling responses in periodontal tissues [24,25,33].

At the tissue level, the failure-relevant consequence is ECM turnover biased toward degradation. Increased expression of matrix metalloproteinases (MMPs) and reduced balance of their inhibitors (TIMPs) can accelerate collagen fragmentation, disrupt cross-links, and degrade proteoglycans that maintain hydration and lubrication. Because proteoglycans and interstitial fluid contribute to load sharing and energy dissipation, their loss increases inter-fibrillar shear and friction, which can further accelerate creep and incomplete recovery. Thus, sustained loading can plausibly create a feed-forward loop: microstrain—mechanotransduction/inflammation—ECM degradation—reduced stiffness/viscoelastic recovery—faster creep accumulation and earlier creep failure [37,38].

Importantly, this hypothesis is not based solely on general connective tissue mechanobiology. Periodontal-specific studies provide direct support that mechanically stimulated human gingival fibroblasts and periodontal fibroblasts alter extracellular matrix turnover, including collagen-related gene expression and MMP/TIMP balance, under sustained or cyclic strain. These findings provide a tissue-relevant cellular basis linking prolonged tensile microstrain to progressive weakening of gingival connective tissue and reduced mechanical recovery capacity in the periodontium [27,31,32,37,38] (see Table 1).

### 2.3. Thin Periodontal Phenotype as a High-Creep State

Although direct in vivo creep–fatigue measurements in human gingiva remain limited, clinical observations from orthodontic tooth movement and phenotype-based recession studies are consistent with the view that sustained low-level mechanical exposure may contribute to marginal soft tissue instability, particularly in thin periodontal phenotypes [1,3,39,41,42,43,44]. The thin periodontal phenotype is characterized by a reduction in gingival connective tissue volume, diminished ECM density, reduced collagen cross-linking, and a thinner facial cortical plate. These anatomical characteristics translate directly into altered mechanical behavior, rendering gingival tissues more prone to viscoelastic deformation and structural fatigue under loading. As a result, thin phenotypes operate closer to the creep failure threshold, even under mild or clinically acceptable orthodontic forces [2,3,41].

Histologic and imaging studies demonstrate that thin phenotypes contain fewer collagen fibers, less organized fiber bundles, and reduced interstitial hydration, all of which decrease tensile stiffness and viscoelastic recovery [1]. Lower ECM density accelerates microstrain accumulation and speeds up the transition from reversible to irreversible creep as fibrillar sliding and microdamage occur earlier and at lower load levels.

Gingival tissues rely on proteoglycans and glycosaminoglycans to maintain hydration, lubrication, and shock absorption. Thin phenotypes exhibit reduced water content and altered proteoglycan composition, impairing fluid-dependent load distribution and increasing friction between fibrils [16,17,21]. This state promotes rapid creep progression under sustained tensile microstrain and delays structural recovery.

Microvascular studies indicate that thin periodontal phenotypes show lower perfusion density, which reduces tissue nutrition, impairs ECM turnover, and makes collagen more susceptible to oxidative and mechanical degradation [42]. Mechanical fatigue develops more quickly in tissues with compromised vascularity, paralleling patterns observed in tendons and dermal tissues under compromised perfusion [8].

Thin phenotypes are hypothesized to have lower effective creep resistance and to approach creep failure conditions earlier than thick phenotypes based on their reduced connective tissue volume, altered ECM organization, and potentially lower reparative reserve. Even low-grade tensile forces generated by orthodontic tooth inclination, lip pressure changes, or archform modification may surpass this threshold, triggering progressive creep and marginal soft tissue displacement [2,3,39].

The combination of diminished ECM stiffness, impaired hydration, reduced vascular support, and a lower creep threshold helps explain why thin periodontal phenotypes exhibit higher incidence and severity of gingival recession and why this risk may not be fully explained by peak force magnitude alone [7,43]. From a biomechanical perspective, recession is not solely a consequence of bone loss or inflammation but the visible manifestation of soft tissue viscoelastic fatigue developing more rapidly in thin tissues.

Thus, the thin phenotype should be understood as a biomechanically vulnerable state predisposed to creep-driven instability and requiring phenotype-specific mechanical strategies. The key biomechanical pathways linking periodontal phenotype, tensile microstrain, and viscoelastic creep behavior are summarized in Table 2.

## 3. The Gingival Creep Failure Theory

**Figure 1:**
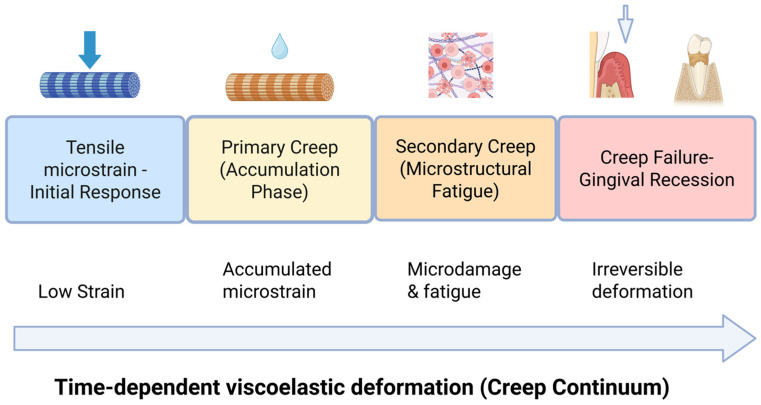
The Gingival Creep Failure Model. Sustained tensile microstrain generates time-dependent viscoelastic deformation in gingival connective tissue. Primary creep involves collagen uncrimping, fibrillar sliding, and fluid redistribution. Secondary creep reflects microstructural fatigue with loss of stiffness and delayed recovery. Once the creep failure threshold is exceeded, irreversible elongation leads to marginal soft tissue displacement and clinical gingival recession. Thin periodontal phenotypes enter the failure zone at significantly lower strain levels.

The Gingival Creep Failure Theory proposes that gingival recession can arise from time-dependent viscoelastic fatigue of soft tissues, even in the absence of excessive orthodontic force or overt inflammation. The theory proposed here is a novel hypothesis introduced by the authors and has not been previously described as a distinct theoretical framework. In this model, gingival connective tissue progressively elongates under sustained or repetitive tensile microstrain, eventually surpassing a structural threshold upon which deformation becomes irreversible. Recession is thus conceptualized as a mechanical failure mode analogous to creep failure in other collagen-based tissues rather than a purely inflammatory or bone-driven phenomenon (see Figure 1). This concept is grounded in established biomechanical evidence demonstrating time-dependent viscoelastic creep, collagen fiber fatigue, and microstructural damage accumulation in collagen-rich connective tissues under sustained loading, including tendons, ligaments, and the periodontal ligament [5,14,45,46].

### 3.1. Sustained Tensile Microstrain Drives Creep Accumulation

Orthodontic and perioral forces (e.g., tooth inclination, soft tissue tension, lip pressure) are hypothesized to generate persistent low-level tensile strain along the gingival margin. Mechanical stimulation of human gingival fibroblasts has been shown to modulate ECM gene expression and matrix turnover (including collagens and MMP/TIMP balance), providing a plausible cellular link between sustained microstrain and progressive loss of connective tissue stability [37,38].

Although each load cycle is insufficient to cause immediate damage, the viscoelastic nature of the ECM causes microstrain to accumulate over time through progressive collagen fiber uncrimping, interfibrillar sliding, and matrix hydration shifts [19,20,21,47]. When exposure is chronic, the tissue gradually transitions from reversible elongation to permanent set, altering vertical and horizontal soft tissue position.

### 3.2. Microstructural Fatigue as the Determinant of Irreversible Margin Displacement

Creep progression accelerates once microdamage accumulates within collagen fibrils and cross-links. Studies on biological soft tissues demonstrate that microfibril fatigue and proteoglycan disruption significantly reduce recoil capacity and increase permanent deformation under sub-threshold load [8,20]. This supports the concept that gingival collagen does not fail instantaneously but undergoes fatigue-driven degeneration, making the marginal tissue increasingly susceptible to displacement even with mild loading.

### 3.3. Creep Failure Threshold Is Phenotype-Dependent

Thin periodontal phenotypes are hypothesized to approach creep failure conditions earlier due to low ECM density, diminished hydration, and reduced vascular support. From a molecular and structural standpoint, periodontal phenotypes differ not only in thickness but also in ECM composition, architecture, and repair capacity. Thin phenotypes are commonly associated with lower collagen volume fraction and altered collagen organization (e.g., smaller or less bundled fibers), which reduces tensile load-bearing capacity and increases fibrillar sliding under sustained load. Differences in collagen cross-link density and quality (including enzymatic cross-links and glycation-related changes) further modulate interfibrillar shear resistance and the ability to recover after deformation. In addition, reduced proteoglycan/GAG (glycosaminoglycans) content and altered hydration decrease lubrication and fluid-mediated load sharing, increasing frictional dissipation between collagen fibrils—conditions that accelerate viscoelastic creep and microdamage accumulation [48]. Phenotype-related differences in microvascular density and baseline inflammatory tone provide an additional biological lever; lower perfusion can reduce ECM turnover and microdamage repair, enabling fatigue-related structural defects to accumulate faster under the same mechanical exposure [12,49].

Together, these phenotype-specific features provide a molecular basis for a lower creep–fatigue tolerance boundary in thin periodontal phenotypes and explain why clinically acceptable orthodontic loads may produce recession in thin tissues while thick phenotypes remain stable [13].

Recent clinical evidence also supports the view that thin gingival phenotype, age, and periodontal inflammatory status are associated with increased recession risk after orthodontic treatment [40].

Mechanical fatigue develops more rapidly in tissues with compromised structure, mirroring observations in tendon, ligament, and dermal tissues where low collagen volume fraction sharply reduces creep resistance [23,41,42,50]. This explains clinical observations that thin phenotypes experience recession under gentle forces while thick phenotypes remain stable.

At present, the creep failure threshold should be regarded as a conceptual boundary rather than a measurable clinical variable. However, future studies may approximate this threshold using surrogate indicators such as progressive marginal displacement on serial intraoral scans or standardized photographs, local reductions in gingival tissue stiffness assessed through elastography, OCT-based evidence of altered deformation behavior, and biologic signatures of ECM turnover, such as sustained shifts in MMP/TIMP balance, collagen disorganization, or altered proteoglycan-related hydration markers. Such surrogates would not directly define the threshold but could provide an empirically tractable approximation of tissue proximity to failure.

### 3.4. Integration with Bone and Phenotype Biology

Importantly, creep-driven soft tissue displacement may be observed even when bone dehiscence is minimal or not radiographically evident, and recession may precede or accompany crestal bone changes rather than be exclusively caused by them, particularly in phenotypes with low viscoelastic resilience. This aligns with studies showing that gingival thinning, collagen degradation, and ECM microdamage can destabilize the margin even when the underlying bone remains intact [43]. Thus, the Gingival Creep Failure Theory provides a biomechanical explanation for recession cases that cannot be attributed to bone integrity or inflammation alone.

A possible interaction with osseous resorption should also be considered. In the present model, bone loss is not treated as the sole initiating mechanism of recession, but it may lower the structural support available to the gingival margin and thereby reduce the effective threshold for creep-related failure. Conversely, progressive soft tissue deformation may expose a pre-existing dehiscence or make subsequent osseous remodeling more clinically evident. The relationship is therefore likely bidirectional: diminished osseous support may facilitate creep accumulation, while creep-driven marginal migration may unmask or amplify the clinical expression of crestal bone loss.

**Figure 2:**
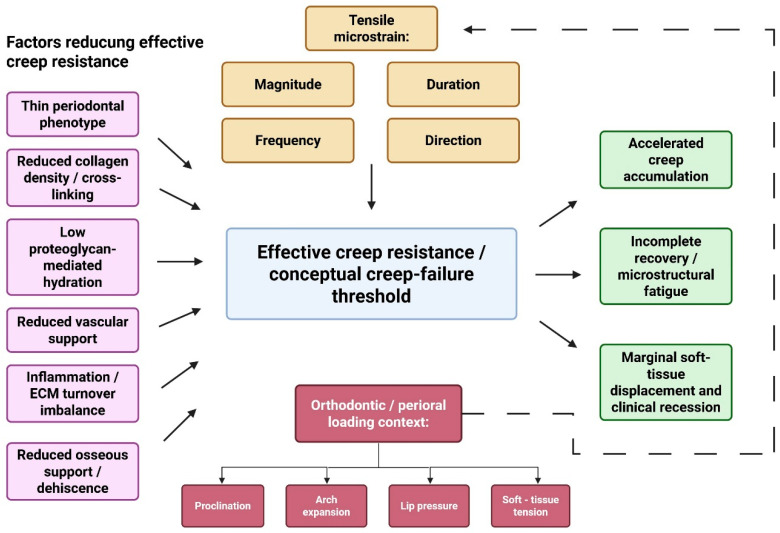
Interacting determinants of gingival creep resistance and creep failure threshold. The diagram summarizes the proposed interaction between tensile microstrain, periodontal phenotype, extracellular matrix quality, hydration, vascular support, inflammation, and osseous support in determining the effective creep resistance of gingival tissues. In this framework, reduced tissue resistance may accelerate creep accumulation, microstructural fatigue, incomplete recovery, and eventual marginal soft tissue displacement. The figure is intended as a hypothesis-organizing model rather than a quantitative representation of validated thresholds.

The proposed interaction between loading variables, tissue quality, inflammation, and osseous support in determining effective creep resistance is summarized in Figure 2.

### 3.5. Clinical Meaning of the Model

The theory predicts that tissues subjected to chronic tensile microstrain may progressively deform as time-dependent strain accumulation and microdamage reduce recoil capacity. Once a tolerance boundary is exceeded, recovery may become incomplete, and the likelihood of clinically detectable recession increases, even when forces remain within clinically accepted ranges.

## 4. Discussion

### 4.1. Direct Evidence Relevant to the Model

The strongest direct evidence supporting the Gingival Creep Failure Theory does not yet come from in vivo demonstration of gingival creep distance but from tissue-relevant observations showing that gingival and periodontal fibroblasts undergo load-dependent changes in extracellular matrix turnover, inflammatory signaling, and vascular response. Mechanical stimulation of human gingival fibroblasts and periodontal fibroblasts modulates collagen-related gene expression, MMP/TIMP balance, inflammatory mediators, and blood-flow-related responses, indicating that sustained loading can alter the material properties and remodeling behavior of periodontal soft tissues [24,25,33,34,35,36,37,38]. These findings do not prove gingival creep failure in vivo, but they do provide direct biologic evidence that the tissues implicated in recession are mechanically responsive and capable of progressive matrix remodeling under load.

### 4.2. Indirect Supporting Evidence from Comparable Tissues

Additional support comes from collagen-rich comparator tissues, such as tendon, ligament, dermis, and periodontal ligament tissues, in which sustained or cyclic sub-failure loading produces viscoelastic deformation, incomplete recovery, collagen molecular unfolding, and fatigue-related loss of mechanical integrity [8,19,20,21,23,41,42,50]. These tissues are not equivalent to gingiva, which is a specialized oral mucosal connective tissue with distinct vascularity, attachment, and exposure to oral loading. However, they provide a biologically relevant proof of principle that time-dependent deformation and creep–fatigue phenomena can arise in hydrated collagenous matrices under clinically modest but repeated loading histories.

### 4.3. Speculative Mechanistic Extensions

Several central elements of the present theory remain hypothetical and should be interpreted as such. These include the magnitude and duration of tensile microstrain required to induce gingival creep in vivo, the existence and location of a clinically meaningful creep failure threshold, the applicability of classical primary/secondary creep phases to gingival tissue, and the quantitative contribution of phenotype, inflammation, hydration, and osseous support to creep resistance [13,16,18,42,48,49]. These components are proposed as mechanistically plausible extensions intended to guide future validation studies rather than as established quantitative facts.

### 4.4. Testable Predictions and Translational Roadmap

The Gingival Creep Failure Theory yields several testable predictions that distinguish viscoelastic fatigue-driven recession from traditional bone- or inflammation-centered paradigms. These predictions provide a framework for experimental validation and clinical translation.

Prediction 1—Recession can occur under light or clinically acceptable forces if tensile microstrain is sustained long enough.

If creep accumulation rather than peak strain drives recession, then even mild, continuous tensile loading—such as lip pressure, proclination, or archwire expansion—should produce progressive deformation when applied over extended periods. Clinical data showing recession developing during “light-force” orthodontic mechanics are broadly consistent with this prediction [1,2,3,39].

Prediction 2—Thin periodontal phenotypes will reach creep–fatigue tolerance limits earlier than thick phenotypes.

Thin gingival tissues with reduced connective tissue volume and altered ECM organization are hypothesized to have reduced viscoelastic tolerance and therefore accumulate creep-related deformation more rapidly. This predicts earlier and more severe recession in thin phenotypes compared with thick phenotypes, consistent with clinical observations linking thin phenotype parameters to higher recession susceptibility [3,42,43].

Prediction 3—Creep-driven migration may be detectable before overt radiographic bone loss or marked inflammatory changes.

If marginal instability is driven by time-dependent soft tissue deformation, early gingival margin migration could be detected in susceptible phenotypes before clear radiographic bone changes or pronounced inflammatory burden. This prediction can be tested using longitudinal standardized photography/scans and imaging-based phenotype assessment [51,52].

Prediction 4—Recovery after load removal will be incomplete once microdamage exceeds viscoelastic tolerance.

Viscoelastic tissues exhibit partial recovery under low creep loads, but once microdamage accumulates, recovery becomes incomplete. The theory predicts that gingival margin recovery after orthodontic force reduction will be minimal in tissues that have already crossed the creep failure threshold [20,23].

Prediction 5—ECM-quality modifiers (age, inflammation, systemic health) will influence recession susceptibility.

Factors that affect collagen cross-linking, hydration, or ECM turnover, such as aging, diabetes, smoking, and chronic inflammatory states, should lower creep resistance and increase recession risk even without high mechanical load [18,53,54,55].

This prediction aligns with epidemiological evidence linking recession to systemic and lifestyle modifiers of connective tissue integrity. Mechanistically, these modifiers converge on collagen quality, proteoglycan-mediated hydration, and the inflammatory MMP/TIMP balance, all of which are known to shift soft tissue viscoelasticity toward greater creep and reduced recovery. Therefore, we expect these factors to act as “ECM quality multipliers” that lower the creep failure threshold even when orthodontic forces remain low [56,57,58].

Prediction 6—Interventions that improve ECM stiffness or hydration should raise the creep failure threshold.

If creep is the underlying mechanism, then modalities that enhance collagen quality or hydration, such as connective tissue grafting, biomaterial scaffolds, or regenerative biologics, should increase the viscoelastic tolerance of the gingiva and improve marginal stability. This prediction is supported by clinical data showing that augmented phenotypes exhibit significantly greater resistance to recession [48,59,60].

In biological terms, phenotype-modifying interventions likely increase collagen volume fraction, improve fiber organization, and alter hydration and vascular support, thereby increasing effective stiffness and viscoelastic recovery. Within this theoretical framework, the expected clinical signature of a successful intervention is not only increased thickness but also reduced time-dependent marginal migration under comparable loading histories.

### 4.5. Clinical Implications

The Gingival Creep Failure Theory reframes gingival recession as a time-dependent mechanical fatigue phenomenon rather than a direct consequence of excessive force or bone loss. This shift in understanding has several clinical implications.

If creep accumulation—not peak stress—is responsible for recession, then long-lasting tensile microstrain poses a greater risk than transient forces. Mechanically valid but chronically sustained orthodontic tension, such as that produced by proclination, expansion, or inadequate lip support, may be sufficient to induce creep-driven tissue deformation [47].

Thin periodontal phenotype patients may exhibit lower ECM stiffness, reduced hydration, and impaired viscoelastic recovery, potentially making them more vulnerable to creep-driven failure [7,8,42]. Pre-treatment phenotype evaluation (gingival and bone thickness) may therefore help inform biomechanical planning and the possible need for soft tissue augmentation.

Orthodontic strategies that minimize constant tensile load—such as intermittent force delivery, reduced proclination angles, or controlled archform expansion—may plausibly reduce creep progression. This aligns with biomechanical studies showing that reducing sustained strain slows viscoelastic fatigue [19,20,21].

Connective tissue grafts or biomaterial scaffolds may increase connective tissue volume, hydration, and stiffness, thereby raising the creep–fatigue tolerance of the gingiva. Clinical evidence and consensus recommendations support phenotype modification as a strategy to improve marginal stability in susceptible cases [59].

Aging, diabetes, and low-grade systemic inflammation diminish collagen cross-linking and hydration, lowering creep resistance [59]. In such patients, even mild strain trajectories may lead to creep-driven recession unless mechanics are modified.

From a translational perspective, the Gingival Creep Failure framework helps explain why certain orthodontic loading configurations disproportionately predispose thin phenotypes to marginal tissue breakdown. Recent mechanobiological models supported by finite element analysis and proof-of-concept CBCT have demonstrated that tension-dominant loading patterns can preserve buccal periodontal tissues by reducing sustained compressive exposure and improving local perfusion. Such approaches may therefore indirectly lower the risk of time-dependent gingival creep failure by limiting the mechanical conditions that promote prolonged tensile microstrain accumulation in soft tissues [61,62].

### 4.6. Future Directions

The Gingival Creep Failure Theory suggests multiple avenues for translational research.

New imaging modalities (e.g., Optical Coherence Tomography (OCT), ultrasound elastography) could allow for dynamic measurement of gingival displacement under loading. Establishing creep curves in real tissues would provide direct validation of the theory. Controlled ex vivo or finite-element models can quantify how much sustained tensile microstrain leads to irreversible deformation in thin versus thick phenotypes. Such data could establish clinical creep failure thresholds. Biologic or scaffold-based therapies that improve collagen density, hydration, or cross-linking may increase gingival creep tolerance. Early evidence suggests that regenerative matrices can improve mechanical resilience and marginal stability [47]. Coupling viscoelastic models with orthodontic FEM (finite element modeling) simulations may predict soft tissue creep trajectories and identify high-risk strain zones around roots and papillae [18,22,63,64]. Tracking gingival displacement over time in different phenotypes and force systems will help determine how load duration, direction, age, and systemic health influence creep progression. Because viscoelastic fatigue is relevant to peri-implant mucosa, prosthetic design, and periodontitis-related soft tissue collapse, the theory may unify diverse clinical observations across oral soft tissue biology.

### 4.7. Limitations and Boundaries

This manuscript presents a conceptual, hypothesis-driven framework and does not provide new in vivo mechanical measurements of gingival creep or fatigue. Direct quantitative creep–fatigue curves for human gingiva and validated in vivo microstrain estimates are currently limited and represent key priorities for future research. The proposed pathway is not intended to exclude established contributors to recession (e.g., inflammation, brushing trauma, bone morphology) but rather to introduce a time-dependent soft tissue mechanical mechanism that may interact with these factors, particularly in thin phenotypes.

A major limitation of the present framework is that sustained tensile microstrain has not yet been quantified in vivo in human gingiva, which precludes calibration of quantitative strain duration thresholds.

## 5. Conclusions

The Gingival Creep Failure Theory proposes a new mechanistic interpretation of gingival recession in which viscoelastic creep and microstructural fatigue may contribute to marginal soft tissue instability, particularly in thin or biologically compromised phenotypes. Rather than replacing established contributors, such as inflammation, brushing trauma, or bone morphology, the model introduces a time-dependent soft tissue mechanical pathway that may interact with these factors during orthodontic treatment and other chronic loading conditions.

By integrating viscoelastic tissue mechanics, periodontal phenotype biology, and orthodontic loading patterns, this theory offers a biologically plausible and clinically relevant hypothesis-generating framework. Its value now lies primarily in organizing observations, generating testable predictions, and guiding future studies aimed at direct quantification of gingival strain, creep accumulation, and tissue-level failure thresholds.

## Figures and Tables

**Table 1:** Gingival- and periodontal-specific evidence supporting the mechanobiology of creep. HGF = human gingival fibroblast; PDL = periodontal ligament; PG = proteoglycan; TLR = Toll-like receptor.

Type of Evidence	Model/System	Loading/Context	Main Observation	Relevance to Gingival Creep Failure Theory	Refs.
In vivo periodontal/gingival loading	Dog gingiva and rat orthodontic tooth movement models	Sustained orthodontic loading (hours to weeks)	Orthodontic force increased gingival MMP-1 activity and altered Col-I/TIMP expression in dog gingiva; pressure-side gingiva in rats showed increased IL-1β, MMP-9, and TIMP-1 expression under orthodontic loading	Supports localized in vivo inflammatory and matrix remodeling responses in periodontal/gingival tissues under mechanical loading	[35,36]
Clinical biomarker evidence	Human gingival crevicular fluid during orthodontic tooth movement	Orthodontic force in vivo (clinical)	Force-associated increases in IL-1β, IL-8, TNFα, MMP-9, TIMP-1/2, and MMP-8 in GCF	Provides human clinical support linking mechanical loading to inflammatory and matrix remodeling responses in the periodontium	[24,25]
Clinical correlation	Thin phenotype/orthodontic recession literature	Human observational data	Recession is more frequent in thin phenotype and in orthodontic settings	Supports phenotype-dependent vulnerability under load	[3,39,40]
Periodontal soft tissue mechanics	Oral soft tissues, oral mucosa, PDL	Ex vivo mechanical testing	Nonlinear site- and time-dependent mechanical behavior	Supports plausibility of time-dependent strain accumulation in periodontal soft tissues	[9,10,23]
Gingival ECM composition	Human gingiva/human fibroblasts	Tissue characterization and ECM regulation	Gingival matrix contains collagen-associated proteoglycans and regulated PG expression	Supports roles of hydration, fibril spacing, and matrix organization in creep recovery	[12,13]
Direct HGF mechanotransduction	HGFs on 3D PLGA scaffold	Applied mechanical force	Integrin α5β1, FAK/p-FAK, COL-1, and stress fibers increase	Shows that gingival fibroblasts actively sense and transduce load	[27]
Direct HGF/PDL remodeling response	HGFs and PDL fibroblasts	Continuous stretch	MMP-1, MMP-2, TIMP-1, TIMP-2, and integrin subunits are altered by strain	Links mechanical loading to ECM turnover and cell matrix adhesion	[37]
Prolonged HGF remodeling	HGFs	Intermittent stretch over 10 days	Collagens I/III/V, elastin, tenascin, proliferation, and cell death are modified	Supports time-dependent shift from adaptation to remodeling/fatigue	[38]
Broader oral soft tissue inflammatory response	Oral mucosa-derived cells, including HGFs	Hydrostatic mechanical stress	Inflammatory cytokine production increases via p38 MAPK	Supports coupling between sustained stress and inflammatory amplification	[28]
Periodontal degradative signaling	Human periodontal fibroblasts	Mechanical force + TLR stimulation	MMP-1, MMP-3, and MMP-10 increase via p38/JNK/NF-κB without parallel TIMP increase	Supports degradative bias under chronic/adverse loading conditions	[31]
Periodontal cell stress signaling	PDL fibroblasts	Mechanical stretch	NF-κB, IL-1β, and apoptosis-related genes are upregulated	Supports load-induced inflammatory and cell stress programs in the periodontium	[32]
Mechanosensitive nuclear signaling in the periodontium	PDL cells	Force-responsive model	YAP-related zyxin modulation is observed	Supports mechanosensitive transcriptional regulation within periodontal tissues	[29]

**Table 2:** Mechanistic links between periodontal phenotype, tensile microstrain, viscoelastic creep behavior, and predicted clinical outcomes.

Factor	Biomechanical Mechanism	Effect in Thin Periodontal Phenotype	Predicted Clinical Manifestation	Key Supporting Evidence
**Collagen fiber density and organization**	Reduced load sharing capacity and accelerated collagen uncrimping and interfibrillar sliding under sustained tensile microstrain	Lower collagen volume fraction and less organized fiber bundles reduce resistance to creep deformation	Earlier onset of irreversible gingival margin displacement under light sustained forces	[1,4,6,8,19]
**Collagen cross-linking quality**	Decreased microstructural stability increases susceptibility to fatigue-related damage during repetitive loading	Reduced cross-link density lowers viscoelastic yield point and accelerates transition from reversible to permanent deformation	Increased susceptibility to recession despite clinically acceptable force magnitude	[4,5,18,19]
**Proteoglycan content and hydration**	Impaired fluid-mediated load distribution and delayed recovery due to altered ECM hydration	Reduced proteoglycan content limits lubrication and shock absorption, promoting strain accumulation	Progressive apical migration of gingival margin under chronic tensile load	[14,15,17,21]
**Vascular support and tissue perfusion**	Reduced metabolic support limits ECM turnover and repair of microdamage	Lower microvascular density accelerates accumulation of fatigue-related microstructural damage	Reduced capacity for tissue recovery after orthodontic force application	[8,42]
**Duration of tensile microstrain**	Time-dependent viscoelastic creep leads to cumulative strain even under low stress levels	Thin phenotypes reach creep failure threshold earlier under sustained loading	Recession develops during prolonged orthodontic treatment with light forces	[3,19,20,39]
**Magnitude vs. duration of orthodontic load**	Sub-failure cyclic or sustained loading induces cumulative deformation rather than acute failure	Thin phenotype is more sensitive to load duration than peak force magnitude	Gingival recession without radiographic bone dehiscence or excessive force	[2,3,45]

## Data Availability

No new data were generated or analyzed in this study.
